# Supplementary material for: Bioinspired nondissipative mechanical energy storage and release in hydrogels via hierarchical sequentially swollen stretched chains
Source: Nat Commun. 2025 May 15;16:4544. doi: 10.1038/s41467-025-59743-w (PMC12081744; doi:10.1038/s41467-025-59743-w)
Supplement: Supplementary file 1 — Supplementary Information [file 41467_2025_59743_MOESM1_ESM.pdf]

Supplementary Information

Bioinspired nondissipative mechanical energy storage and release in hydrogels via hierarchical sequentially swollen stretched chains

Henri Savolainen[1], Negar Hosseiniyan[1], Mario Piedrahita-Bello[1], Olli Ikkala[1*]*

[1]Department of Applied Physics, Aalto University, 02150 Espoo, Finland

**Corresponding Authors: M.P., O.I.*

E-mail: mario.piedrahitabello@aalto.fi olli.ikkala@aalto.fi

Determination of Energy Storage Efficiency

The energy storage efficiency was determined graphically by converting other publication cyclic tensile test graphs into shapes which cover the area below the curves. Then, the size of the curves were analyzed via ImageJ. Stored energy in our own cyclic tensile tests were determined by numerical approximation via the trapezoid method.

Determination of Energy density

We start with the neo-hookean model [1] $\sigma = \frac{E}{3}\left(\lambda - \frac{1}{\lambda^2}\right)$ and integrate by λ to gain the energy density $U = \frac{E}{3}\left(\frac{\lambda^2}{2} + \frac{1}{\lambda}\right)$. As biological systems operate in small strain ranges, the neo-hookean model is applicable[2-4].

Young's Modulus Determination of the literature

Either publications stated the Young's Modulus or a linear fit was done to the cyclic tensile tests at small strains and therefore determined graphically

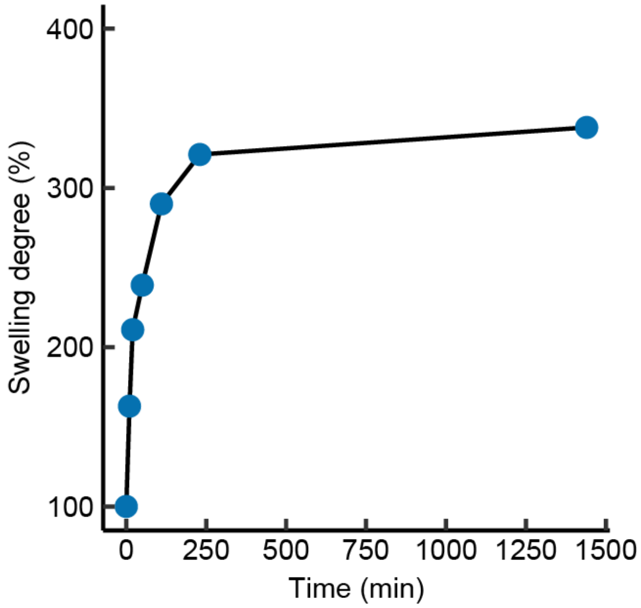

Supplementary Figure 1: **Swelling degree.** Swelling degree as a function of time of a sample of PAAm hierarchical gel at a hierarchical level N = 2.

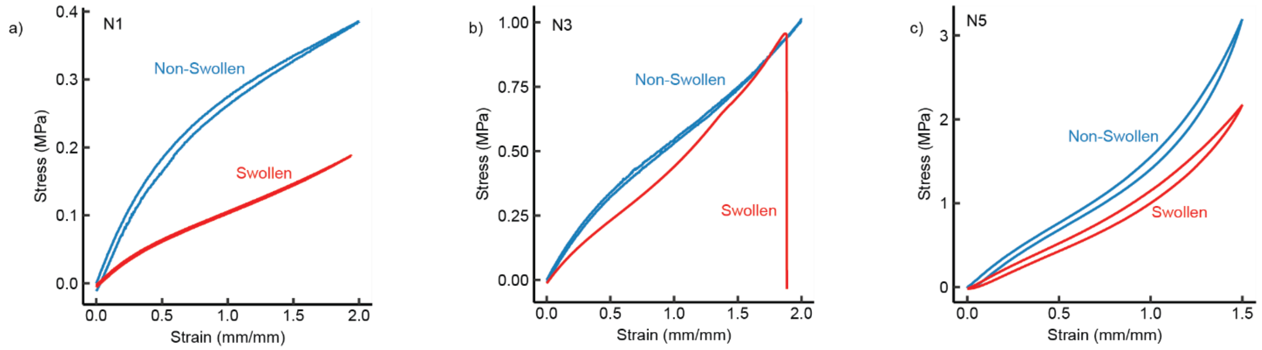

Supplementary Figure 2: Comparison of swollen and nonswollen PAAm hydrogels. **a**, Cyclic tensile test for N = 1. **b**, Cyclic tensile test for *N* = 3. c) Cyclic tensile test for *N* = 5.

Supplementary Table 1: Mechanical properties of PAAm based hierarchically stretched hydrogels. Measurements were done at 60% air humidity.

Level	Young's Modulus [MPa]	Ultimate Tensile Strength [MPa]	Maximum Strain [mm/mm]	Cyclic Strain [mm/mm]	Maximum cyclic stress [MPa]	Stored Energy [MJ/m^3]	Energy Storage Efficiency (%)
***N* = 1**	0.596 +- 0.065	0.679 +- 0.74	4.38 +- 0.78	2	0.387+- 0.044	0.487 +- 0.049	6+-1.18
***N* = 2**	0.818 +- 0.055	0.957 +- 0.201	2.32 +- 0.51	2	0.726+- 0.052	0.83 +- 0.037	3.33+- 2.47
***N* = 3**	0.1.033 +- 0.112	1.499 +- 0.691	2.34 +- 0.48	2	0.960 +- 0.036	1.024+- 0.019	2.5+- 0.76
***N* = 4**	1.157 +- 0.188	1.935 +- 0.830	2.67 +- 0.77	1.5	1.648+- 0.451	1.021+- 0.136	8.4+- 3.06
***N* = 5**	1.836 +- 0.253	4.541 +- 1.697	1.71 +- 0.41	1.5	3.100 +- 0.792	1.625 +- 0.239	11.85+- 4.05
***N* = 6**	3.523 +- 0.536	4.334 +- 1.420	0.91 +- 0.21	1	3.921 +- 0.396	1.275 +- 0.111	16.7+- 0.5
***N* = 7**	4.817 +- 1.416	5.249 +- 2.391	1.28 +- 0.47	1	4.798 +- 0.339	1.248 +- 0.081	50.8+- 2.6

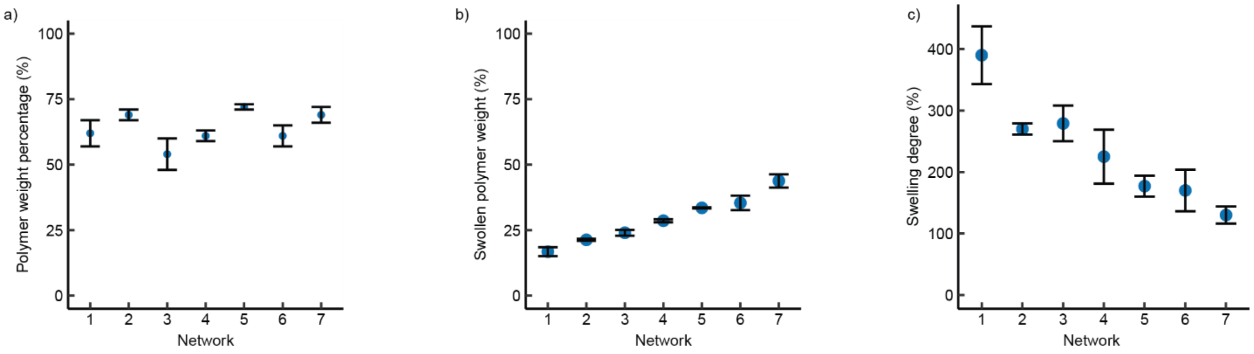

Supplementary Figure 3: Polymer weight percentage analysis for hierarchically swollen PAAm hydrogels upon hierarchies *N*. **a**, Polymer weight percents **b**, Polymer weight percent in the swollen state. **c**, Swelling degree by number of networks in monomer solution.Values displayed are averages of at least n=3 samples and error is given in standard deviation.

The swelling degree is defined as follows

$$\rho = \frac{weight_{swollen}}{weight_{pristine}}$$

The polymer weight % is defined as follows

$$\rho = \frac{weight_{dry}}{weight_{pristine}}$$

The swollen polymer weight % is defined as follows

$$\rho = \frac{weight_{dry}}{weight_{swollen}}$$

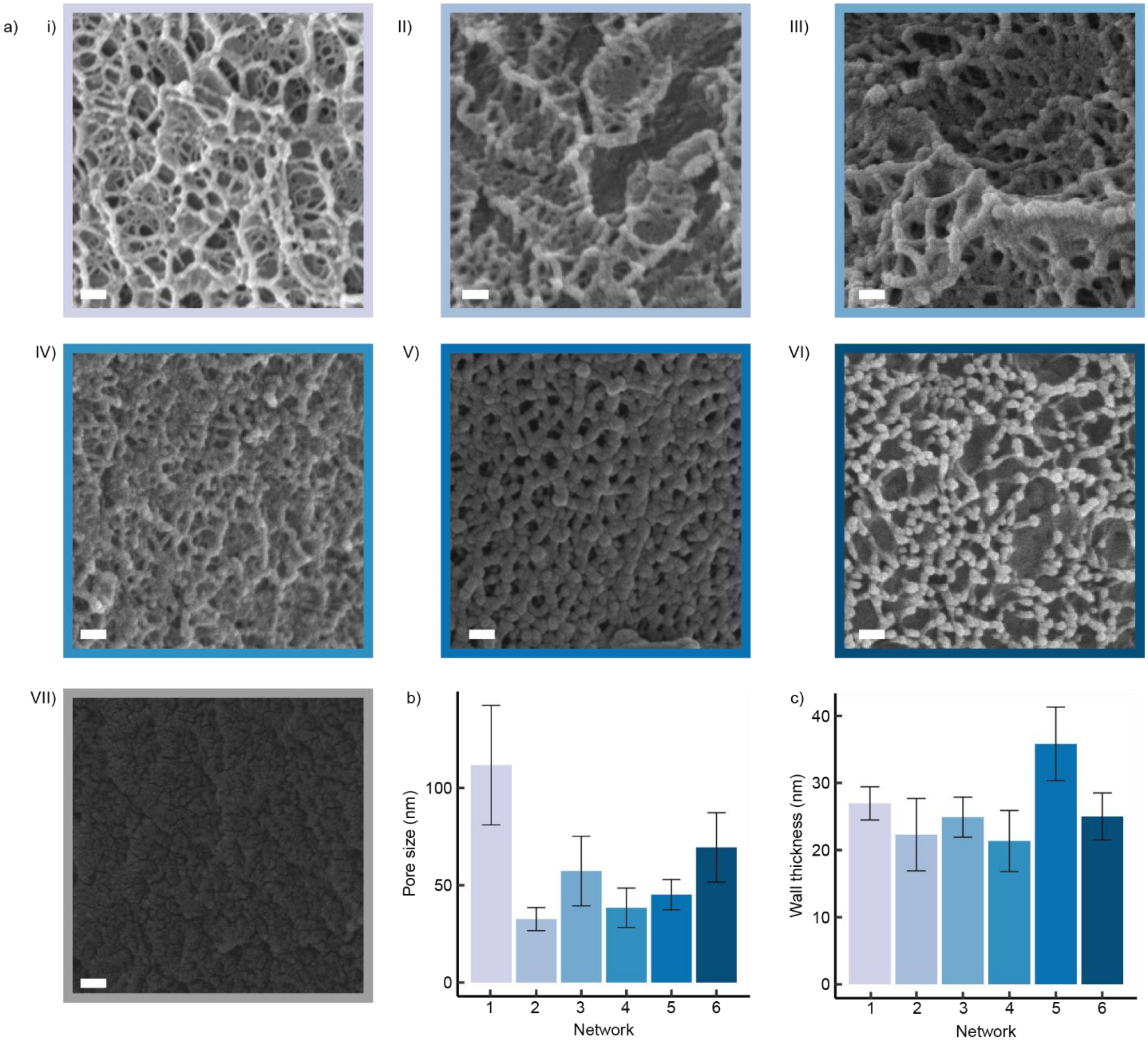

Supplementary Figure 4: Networks of hierarchical swollen PAAm hydrogels for hierarchical levels from *N* = 1 to 7. a, SEM micrographs as a function of *N*. Scalebar represents 100 nm. **b,** Pore sizes as a function of *N*. **c,** Wall thickness as a function of *N*. Values are displayed as averages of n=10 points and errorbars calculated by standard deviation.

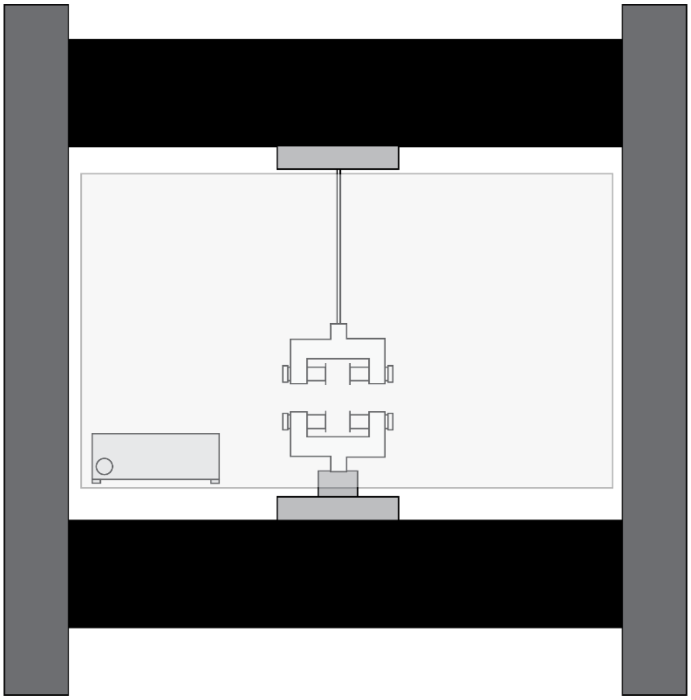

Supplementary Figure 5: Experimental setup. Schematics of the experimental setup for long term mechanical cyclic testing of samples with a chamber and humidifier.

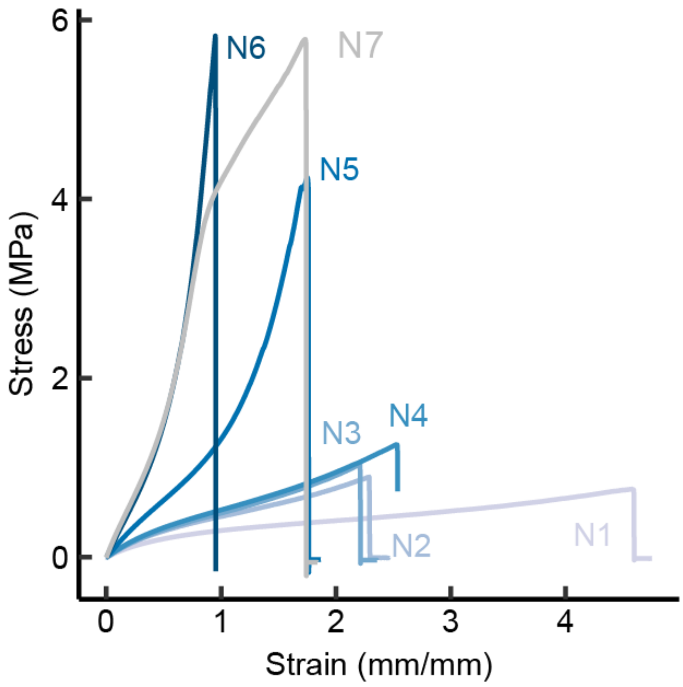

Supplementary Figure 6: Tensile tests. Tensile tests of hierarchically swollen PAAm hydrogels for N = 1 to 7.

Supplementary Table 2: Data of the literature comparison.

Polymer	Tensile Strength [kPa]	Young's Modulus [kPa]	Energy Storage Efficiency (%)	Source
**PAAm**	20	15	95	[5]
**PAAm**	36	16	98	[6]
**PAAm**	150	60	94	[7]
**PEG**	75	400	100	[8]
**HEC**	150	200	100	[8]
**PAAm**	85	100	100	[9]
**PEG/PDMS**	65	35	98	[10]
**PVP**	120	65	98	[11]
**PAAm**	110	60	100	[12]
**PAAm/PEG**	120	100	96	[13]
**PAAc/PAAm**	130	100	98	[14]
**PAAm**	600	100	87	[15]
**Natural resilin**	2942	640	90	[16,17]
**PAAm+CaCl2**	450	30	99	[18]
**PAAm**	40	37	99	[19]
**PAAm/Alginate**	8	4	95	[20]
**PEG**	40	10	100	[21]
**Silicone**	400	150	97	[22]
**PEG**	1750	130	100	[1]
**PVA**	30	60	100	[23]
**PAAm**	16	7.3	100	[24]
**PEG**	1000	60	90	[25]
**PAMPS/PAAm**	275	72	100	[26]
**Natural Elastin**	1500	1000	92	[17,27]

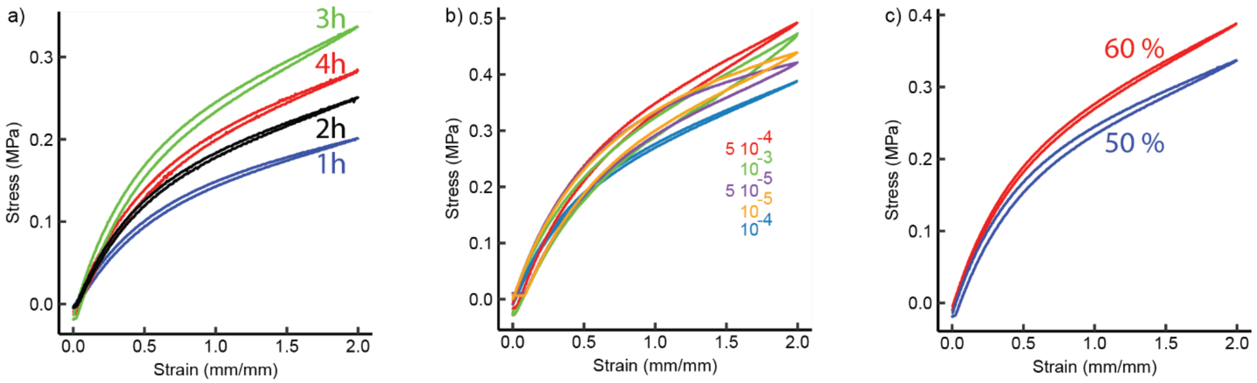

Supplementary Figure 7: Parameter optimization of hierarchically swollen PAAm hydrogels. a, Optimization of irradiation time on samples with 50 wt. % and 10^{-4} mol % crosslinker (BIS). **b,** Optimization of BIS concentration of 60 wt. % samples. **c,** Optimization of polymer weight percentage with 10^{-4} mol% BIS.

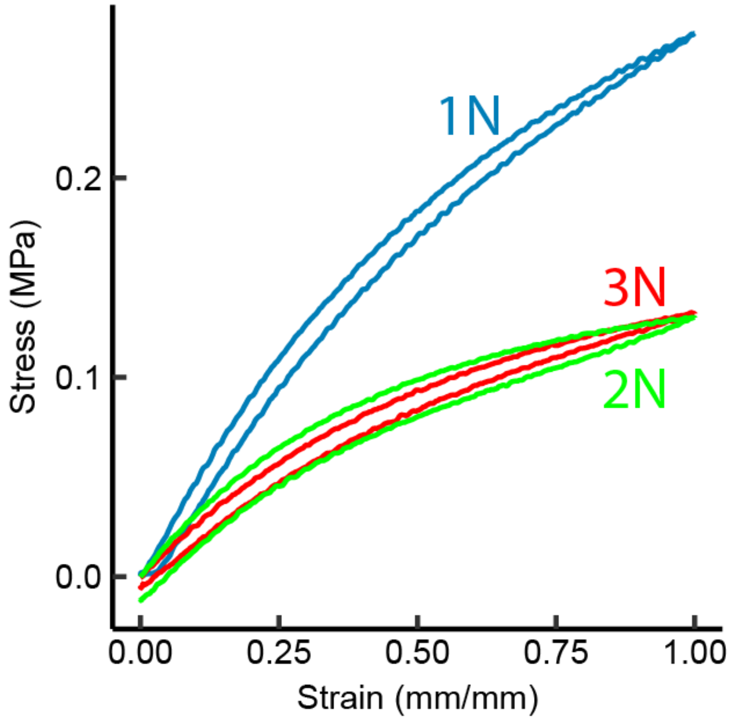

Supplementary Figure 8: Exploration of samples without crosslinkers and 60 wt.%. Each step swollen for 1 hour and then irradiated for 1 hour.

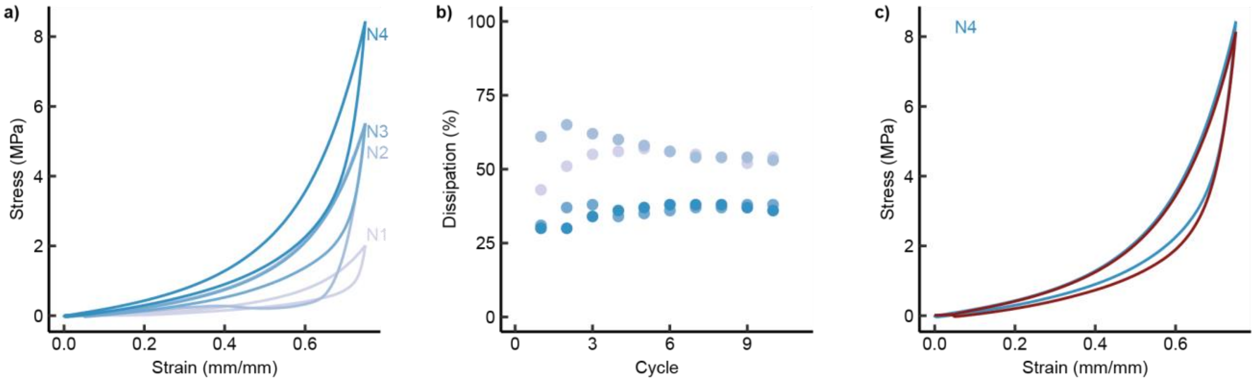

Supplementary Figure 9: Compression tests. **a,** Cyclic compression of samples *N* = 1 to 4 to a strain of 0.75. **b,** Dissipation of multiple cyclic tests for samples of *N* = 1 to *N* = 4. **c**, The first and tenth cyclic compression test of *N* = 4.

Supplementary Table 3: Mechanical properties of PNIPAm, POEGMA, PDMAA, and PAAc based hierarchically swollen hydrogels.

Polymer	Stage	Cyclic strain [mm/mm]	Maximum cyclic stress [kPa]
**PNIPAm**	1	3	24.2 ± 1.2
**PNIPAm**	2	3	57.6 ± 18.3
**PNIPAm**	3	3	70.4 ± 21.8
**POEGMA**	1	0.5	12.8 ± 1.06
**POEGMA**	2	0.5	19.2 ± 1.7
**POEGMA**	3	0.5	24.3 ± 4.1
**PDMAA**	1	1	72.6 ± 6.8
**PDMAA**	2	1	126.3 ± 23.1
**PDMAA**	3	1	155.8 ± 40.6
**PAAc**	1	1	182.2 ± 9.1
**PAAc**	2	1	295 ± 16.3
**PAAc**	3	1	348.5± 103.5

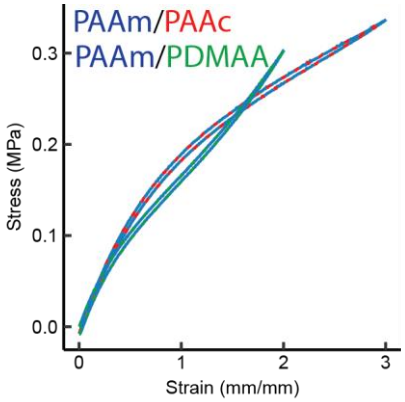

Supplementary Figure 10: Comparison of different polymers in cyclic testing. *N* = 2 samples based on PAAm and then PAAc and samples based on PAAm and PDMAA.

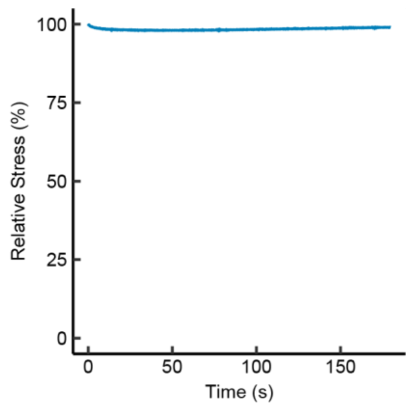

Supplementary Figure 11: Step strain test. Step strain test of PAAm sample of $N = 4$.

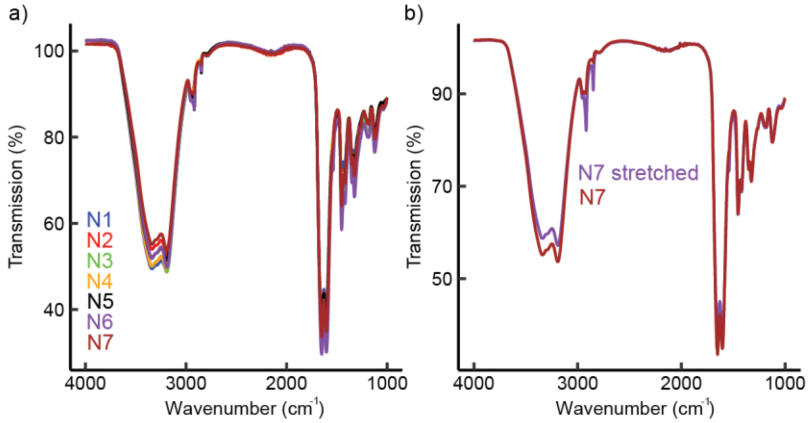

Supplementary Figure 12: Infrared spectra. FTIR of PAAm. **a,** FTIR for *N*1-*N*7. **b,** FTIR of *N*7 stretched and non stretched.

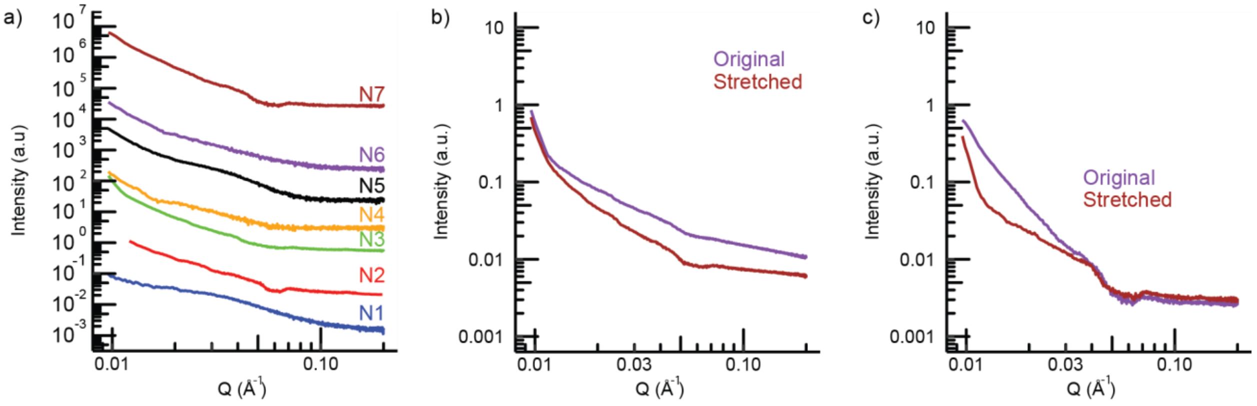

Supplementary Figure 13: SAXS measurements. SAXS measurements. **a,** SAXS of *N* 1-7. **b,** SAXS of swollen *N*6 original and stretched. **c,** SAXS of *N*7 original and stretched.

References:

1 Liu, C. *et al.* Tough hydrogels with rapid self-reinforcement. *Science* **372**, 1078-1081 (2021). https://doi.org/10.1126/science.aaz6694

2 Astley, H. C. & Roberts, T. J. Evidence for a vertebrate catapult: elastic energy storage in the plantaris tendon during frog jumping. *Biol Lett* **8**, 386-389 (2012). https://doi.org/10.1098/rsbl.2011.0982

3 Bennet-Clark, H. C. & Lucey, E. C. The jump of the flea: a study of the energetics and a model of the mechanism. *J Exp Biol* **47**, 59-67 (1967). https://doi.org/10.1242/jeb.47.1.59

4 Kharazi, M., Bohm, S., Theodorakis, C., Mersmann, F. & Arampatzis, A. Quantifying mechanical loading and elastic strain energy of the human Achilles tendon during walking and running. *Sci Rep* **11**, 5830 (2021). https://doi.org/10.1038/s41598-021-84847-w

5 Zhu, L., Zhang, X., Shao, Z. & Guo, M. Highly Stretchable, Compressible, Resilient, and Equilibrium Swelling Hydrogels with Elastic Nano Junctions. *Macromolecular Materials and Engineering* **305** (2020). https://doi.org/10.1002/mame.202000205

6 Ko, S., Chhetry, A., Kim, D., Yoon, H. & Park, J. Y. Hysteresis-Free Double-Network Hydrogel-Based Strain Sensor for Wearable Smart Bioelectronics. *ACS Appl Mater Interfaces* **14**, 31363-31372 (2022). https://doi.org/10.1021/acsami.2c09895

7 Liu, R. *et al.* Highly tough, stretchable and resilient hydrogels strengthened with molecular springs and their application as a wearable, flexible sensor. *Chemical Engineering Journal* **415** (2021). https://doi.org/ARTN 128839

10.1016/j.cej.2021.128839

8 Nian, G., Kim, J., Bao, X. & Suo, Z. Making Highly Elastic and Tough Hydrogels from Doughs. *Adv Mater* **34**, e2206577 (2022). https://doi.org/10.1002/adma.202206577

9 Kim, J., Zhang, G., Shi, M. & Suo, Z. Fracture, fatigue, and friction of polymers in which entanglements greatly outnumber cross-links. *Science* **374**, 212-216 (2021). https://doi.org/10.1126/science.abg6320

10 Cui, J. *et al.* Synthetically simple, highly resilient hydrogels. *Biomacromolecules* **13**, 584-588 (2012). https://doi.org/10.1021/bm300015s

11 He, C. *et al.* Tough and super-resilient hydrogels synthesized by using peroxidized polymer chains as polyfunctional initiating and cross-linking centers. *Soft Matter* **9** (2013). https://doi.org/10.1039/c2sm27605d

12 Wang, Y., Nian, G., Kim, J. & Suo, Z. Polyacrylamide hydrogels. VI. Synthesis-property relation. *Journal of the Mechanics and Physics of Solids* **170** (2023). https://doi.org/10.1016/j.jmps.2022.105099

13 Lei, H. *et al.* Stretchable hydrogels with low hysteresis and anti-fatigue fracture based on polyprotein cross-linkers. *Nat Commun* **11**, 4032 (2020). https://doi.org/10.1038/s41467-020-17877-z

14 Prado, R. M. B. *et al.* Achieving High-Speed Retraction in Stretchable Hydrogels. *ACS Appl Mater Interfaces* **12**, 40719-40727 (2020). https://doi.org/10.1021/acsami.0c08132

15 Li, W. *et al.* Nanoconfined polymerization limits crack propagation in hysteresis-free gels. *Nat Mater* **23**, 131-138 (2024). https://doi.org/10.1038/s41563-023-01697-9

16 Weis-Fogh, T. Thermodynamic properties of resilin, a rubber-like protein. *Journal of Molecular Biology* **3**, 520-531 (1961). https://doi.org/10.1016/s0022-2836(61)80018-1

17 Gosline, J. *et al.* Elastic proteins: biological roles and mechanical properties. *Philos Trans R Soc Lond B Biol Sci* **357**, 121-132 (2002). https://doi.org/10.1098/rstb.2001.1022

18 Wang, J., Tang, F., Yao, C. & Li, L. Low Hysteresis Hydrogel Induced by Spatial Confinement. *Advanced Functional Materials* **33** (2023). https://doi.org/10.1002/adfm.202214935

19 Xu, K. *et al.* Ultradurable Noncovalent Cross-Linked Hydrogels with Low Hysteresis and Robust Elasticity for Flexible Electronics. *Chemistry of Materials* **34**, 3311-3322 (2022). https://doi.org/10.1021/acs.chemmater.2c00093
20 Lin, S., Zhou, Y. & Zhao, X. Designing extremely resilient and tough hydrogels via delayed dissipation. *Extreme Mechanics Letters* **1**, 70-75 (2014). https://doi.org/10.1016/j.eml.2014.11.002
21 Kamata, H., Akagi, Y., Kayasuga-Kariya, Y., Chung, U. I. & Sakai, T. "Nonswellable" hydrogel without mechanical hysteresis. *Science* **343**, 873-875 (2014). https://doi.org/10.1126/science.1247811
22 Si, L. *et al.* Silicone-based tough hydrogels with high resilience, fast self-recovery, and self-healing properties. *Chem Commun (Camb)* **52**, 8365-8368 (2016). https://doi.org/10.1039/c6cc02665f
23 Lin, S. *et al.* Anti-fatigue-fracture hydrogels. *Sci Adv* **5**, eaau8528 (2019). https://doi.org/10.1126/sciadv.aau8528
24 Su, X., Mahalingam, S., Edirisinghe, M. & Chen, B. Highly Stretchable and Highly Resilient Polymer-Clay Nanocomposite Hydrogels with Low Hysteresis. *ACS Appl Mater Interfaces* **9**, 22223-22234 (2017). https://doi.org/10.1021/acsami.7b05261
25 Fujiyabu, T. *et al.* Tri-branched gels: Rubbery materials with the lowest branching factor approach the ideal elastic limit. *Sci Adv* **8**, eabk0010 (2022). https://doi.org/10.1126/sciadv.abk0010
26 Zhu, R., Zhu, D., Zheng, Z. & Wang, X. Tough double network hydrogels with rapid self-reinforcement and low hysteresis based on highly entangled networks. *Nat Commun* **15**, 1344 (2024). https://doi.org/10.1038/s41467-024-45485-8
27 Aaron, B. B. & Gosline, J. M. Elastin as a random-network elastomer: A mechanical and optical analysis of single elastin fibers. *Biopolymers* **20**, 1247-1260 (2004). https://doi.org/10.1002/bip.1981.360200611
