# Supplementary material for: Bioinspired nondissipative mechanical energy storage and release in hydrogels via hierarchical sequentially swollen stretched chains
Source: Nat Commun. 2025 May 15;16:4544. doi: 10.1038/s41467-025-59743-w (PMC12081744; doi:10.1038/s41467-025-59743-w)
Supplement: Supplementary file 2 — Description of Addtional Supplementary File [file 41467_2025_59743_MOESM2_ESM.pdf]

Supplementary Movie 1: **Jumping prototype.** Movie showing, in real time, a soft jumper prototype using hierarchical PAAm gels for mechanical energy storage.
